# The development and validation of the Youth Actuarial Care Needs Assessment Tool for Non-Offenders (Y-ACNAT-NO)

**DOI:** 10.1186/s12888-015-0421-1

**Published:** 2015-03-04

**Authors:** Mark Assink, Claudia E van der Put, Frans J Oort, Geert Jan JM Stams

**Affiliations:** Research Institute of Child Development and Education, University of Amsterdam, Nieuwe Achtergracht 127, 1018 WS Amsterdam, The Netherlands

**Keywords:** Risk assessment, Screening, Actuarial prediction, Police, Juveniles, Care needs, Problematic child-rearing situations, CHAID analysis

## Abstract

**Background:**

In The Netherlands, police officers not only come into contact with juvenile offenders, but also with a large number of juveniles who were involved in a criminal offense, but not in the role of a suspect (i.e., juvenile non-offenders). Until now, no valid and reliable instrument was available that can be used by Dutch police officers for estimating the risk for future care needs of juvenile non-offenders. In the present study, the Youth Actuarial Care Needs Assessment Tool for Non-Offenders (Y-ACNAT-NO) was developed for predicting the risk for future care needs that consisted of (1) a future supervision order as imposed by a juvenile court judge and (2) future worrisome incidents involving child abuse, domestic violence/strife, and/or sexual offensive behavior at the juvenile’s living address (i.e., problems in the child-rearing environment).

**Methods:**

Police records of 3,200 juveniles were retrieved from the Dutch police registration system after which the sample was randomly split in a construction (*n* = 1,549) and validation sample (*n* = 1,651). The Y-ACNAT-NO was developed by performing an Exhaustive CHAID analysis using the construction sample. The predictive validity of the instrument was examined in the validation sample by calculating several performance indicators that assess discrimination and calibration.

**Results:**

The CHAID output yielded an instrument that consisted of six variables and eleven different risk groups. The risk for future care needs ranged from 0.06 in the lowest risk group to 0.83 in the highest risk group. The AUC value in the validation sample was .764 (95% CI [.743, .784]) and Sander’s calibration score indicated an average assessment error of 3.74% in risk estimates per risk category.

**Conclusions:**

The Y-ACNAT-NO is the first instrument that can be used by Dutch police officers for estimating the risk for future care needs of juvenile non-offenders. The predictive validity of the Y-ACNAT-NO in terms of discrimination and calibration was sufficient to justify its use as an initial screening instrument when a decision is needed about referring a juvenile for further assessment of care needs.

## Background

In The Netherlands, a large number of organizations are involved in protecting children from maltreatment. One of these organizations is the Dutch police, as police officers come into contact with large groups of both juvenile offenders as well as juvenile non-offenders (i.e., juveniles involved in an offense, but not in the role of a suspect). For prevention purposes, it is important that police officers can identify serious problems in the juvenile’s rearing environment at an early stage (i.e., problematic family circumstances at the juvenile's living address), so that juveniles can be referred timely to specialized youth care agencies for further assessment and treatment if necessary. The decision about referring a juvenile for further assessment requires that police officers have a valid and reliable screening instrument for estimating the risk for future care needs.

Today, numerous instruments are available for the assessment of risk for future child welfare involvement and/or risk for different types of child maltreatment that children are exposed to in their environment (e.g., see [1,2] for an overview of instruments). However, many of these instruments assess the risk for the recurrence of child maltreatment either among juveniles who experienced substantiated maltreatment or among juveniles who are already under the supervision of child protection services. Instruments that can be used specifically by police officers to screen for the risk for future care needs of juvenile non-offenders are far less available. Furthermore, many instruments that are available require that the assessment is done by specifically skilled, well-trained, and supported professionals in order to be effective [3]. Although the Dutch police plays an important role in the chain of youth care, Dutch police officers do not have the time, resources, and expertise to engage in thorough clinical assessments of large groups of juveniles, making many of these instruments not very well suitable for the task at hand. Therefore, the purpose of the present study was to develop an instrument that can be integrated in the Dutch police system, so that an automatic screening process is possible within a limited time frame and with minimal cost. As a consequence, this instrument had to be developed using only information available in operational police systems. A further aim was to consider the psychometric quality of this instrument by examining its predictive validity in terms of discrimination and calibration.

Recently, an actuarial risk screening instrument (Youth Offender Care Needs Assessment Tool; YO-CNAT) was developed to provide Dutch police officers an indication of the risk for future care needs of juvenile offenders, which is needed for the decision to refer these juveniles for further assessment [4]. This instrument was specifically developed for young offenders and solely based on information available in the Dutch police system, so that it can be used by Dutch police officers who, in general, do not have a clinical background. In addition, the YO-CNAT was designed for implementation in the Dutch police system so that the risk for future care needs can automatically be calculated. The following seven predictor variables are part of the YO-CNAT: the number of official recorded incidents involving (1) domestic disputes, (2) child abuse, (3) sexual offensive behavior, and (4) domestic violence at the juvenile’s living address; (5) age on first recorded incident in which the juvenile had any role (including the role of a suspect); (6) the total number of recorded incidents in which one of the juvenile’s co-occupants was involved as a suspect; and (7) the total number of recorded incidents in which the juvenile was involved (having any role). The items comprising the YO-CNAT are presented in Table 1. Van der Put and Stams [4] showed that the discriminatory accuracy of this instrument ranged between acceptable (with an AUC value of .701) and good (with an AUC value of .745) for predicting different types of future care needs. The YO-CNAT was specifically developed for screening juvenile offenders, but police officers also come into contact with large numbers of juvenile *non*-offenders, who were involved in a criminal incident but not in the role of a suspect. At present, there is no screening instrument available for estimating the risk for future care needs of juvenile non-offenders that can be implemented in the Dutch police system, so that large numbers of juvenile non-offenders can automatically be screened within a limited time frame and with minimal cost.

**Table 1:** **Items comprising the Youth Offender Care Needs Assessment Tool (YO-CNAT)**

**Items**	**Scale**
(1) The number of recorded incidents involving domestic disputes at the juvenile’s living address	□ 0 incidents
	□ 1 or more incidents
(2) The number of recorded incidents involving child abuse at the juvenile’s living address	□ 0 incidents
	□ 1 or more incidents
(3) The number of recorded incidents involving sexual offensive behavior at the juvenile’s living address	□ 0 incidents
	□ 1 or more incidents
(4) The number of recorded incidents involving domestic violence at the juvenile’s living address	□ 0 incidents
	□ 1 or more incidents
(5) The age of the juvenile when he/she was involved in a recorded incident for the first time (having any role)	□ 11 years or younger
	□ 12 or 13 years
	□ 14 or 15 years
	□ 16 years or older
(6) The number of recorded incidents in which a co-occupant at the juvenile’s living address was involved in the role of a suspect	Number of incidents^a^
(7) The number of recorded incidents in which the juvenile was involved (having any role)	Number of incidents^a^

The YO-CNAT is an initial screening instrument that is developed for estimating the risk for future care needs (i.e., the probability of future problematic child-rearing situations) of juvenile offenders [4]. This instrument can only be used by the Dutch police since it was designed for implementation in the Dutch police system.

^a^Different categorizations of these two variables are used in calculating the risk for future care needs and are not presented here. See [4] for more information on the categories.

Initially, it may seem that the YO-CNAT [4] can also be used for estimating the risk for future care needs of juvenile non-offenders, but three important issues can make this problematic. First, not all predictor variables that are part of the YO-CNAT apply to juvenile non-offenders, since these juveniles have never been recorded in the police system as having the role of a suspect. Second, the YO-CNAT can only yield an optimal prediction of risk among juvenile non-offenders if the base rates of care needs among juvenile offenders and juvenile non-offenders would be approximately equal. Although base rates have no impact on sensitivity or specificity, they exert considerable influence on the predictive powers of an instrument. We expect care needs to be more common in juvenile offenders than in juvenile non-offenders because victimization of child maltreatment is positively related to offending behavior [5-11]. Third, the optimal cutoff score that was determined for the YO-CNAT may not be optimal or even appropriate for risk prediction among juvenile non-offenders. Because these issues can contribute to an unsatisfactory performance of the YO-CNAT when it is used for risk prediction among juvenile non-offenders, we believe that it is important to examine whether a valid and reliable risk screening instrument can be developed for specifically juvenile non-offenders.

In sum, the aim of the present study was to develop a valid and reliable screening instrument for the prediction of future care needs of juvenile non-offenders. This instrument will be further referred to as the Youth Actuarial Care Needs Assessment Tool for Non-Offenders (Y-ACNAT-NO). In developing and validating the Y-ACNAT-NO, a stepwise approach was used. First, we examined the extent to which different types of police records were related to future care needs. Second, we examined whether a valid screening instrument could be developed with sufficient predictive power, using only information stored in the Dutch police system. Third, we examined whether this instrument was suitable for the prediction of specific forms of care needs, and fourth, we compared the predictive power of the Y-ACNAT-NO to the predictive power of the YO-CNAT to determine whether the development of a new instrument for predicting future care needs of juvenile non-offenders could be justified.

## Methods

### Sample

The sample consisted of 3,200 juveniles between the ages of 12 and 18 years (*M* = 15.4, *SD* = 1.7), who were registered in official Dutch police records in 2007 because they were involved in an offense, but not in the role of a suspect (i.e., registered as victim, witness, reporting subject, or missing person; registered as a juvenile attracting police attention; or registered as a juvenile having any role not otherwise defined by the Dutch police). These juveniles were selected at random from all juveniles who came into contact with the Dutch police in 2007 in five Dutch police regions. During their lives, the selected juveniles had never been suspected by the Dutch police of committing an offense (i.e., they had never been recorded in the role of a suspect). The incident that occurred in 2007 was regarded as the index incident, and the official records of the random sample of juveniles were retrieved from the computer system of the Dutch police for a period of five years prior to the date of the index incident (i.e., from 2002 to 2007). This period is restricted to five years because the Dutch Police Data Act prescribes that information be stored in police systems for a maximum period of five years.

To construct and validate the instrument, the sample was split randomly into a construction sample (*n* = 1,549) and a validation sample (*n* = 1,651). The size of the full sample (*N* = 3,200) was sufficiently large for splitting this sample into a construction and validation sample to perform an Exhaustive CHAID analysis in which the total group of juveniles was divided into a number of smaller subgroups. No significant differences were found between the construction and validation sample in terms of gender (*χ*^*2*^(1) = .744, *p* = .388), country of birth (*χ*^*2*^(1) = .317, *p* = .573), and age (*t*(3,198) = 1.20, *p* = .230).

### Independent and dependent variables

Before a screening instrument can be developed, it should first be determined which potential risk factors need to be included as predictor variables in the analysis to construct the instrument. Therefore, we performed a literature search on risk factors most consistently associated with child maltreatment. It is important to note that it should not automatically be assumed that both the occurrence and recurrence of child maltreatment can be predicted by the same set of variables [12]. According to Cash [13], the following factors are associated with initial maltreatment: maternal and paternal depression; substance abuse; unemployment; social isolation; unrealistic expectations of the child; parent’s history of being abused; and increased stress. On the other hand, factors that are more associated with the recurrence of maltreatment are: parents’ previous involvement with child protection services; parents’ unrealistic expectations of the child; the child’s level of fear towards the perpetrator and the child’s contact with the perpetrator; neglect; parental conflict; and parental mental health problems [14-16]. In a systematic review, Hindley, Ramchandani, and Jones [17] identified four factors that were most consistently associated with the recurrence of child maltreatment: number of previous episodes of maltreatment; neglect (as opposed to other forms of maltreatment); parental conflict; and parental health problems. Kerig and Wenar [18] also mentioned several personality, socioeconomic, and household characteristics as important predictors of both child maltreatment and domestic violence. In selecting possible predictors from all variables stored in the police system, we tried to match predictors with important risk factors as just described in the best possible way. However, many important risk factors could not be part of the instrument, simply because information pertaining to important risk factors, such as parental (mental) health problems and previous involvement with child protection services, is unavailable to the Dutch police.

The dependent variable (“care needs”) was based on information gathered from both the Dutch Youth Care Agency (“Bureau Jeugdzorg”) and the Dutch police. In The Netherlands, the Youth Care Agency is the first authority in the chain of youth services that is responsible for the assessment of the nature and seriousness of problematic child-rearing situations, and comes into action when a notification of a problematic situation is made. If the Youth Care Agency is indeed concerned about the situation, it notifies the Child Protection Board, which will then investigate whether the juvenile should be placed under supervision. A juvenile court judge is then informed about the results of this investigation and may impose a supervision order that will be carried out by the Youth Care Agency. Therefore, we regarded an imposed supervision order as an indication of a problematic child-rearing situation, and thus as a care need.

On the other hand, there are also juveniles with care needs who do not come to the attention of the Youth Care Agency. Many of these juveniles do come in contact with the Dutch police because of involvement in a worrisome incident of child abuse, domestic violence, and/or sexual offensive behavior (in which the juvenile is not involved as a suspect). These incidents are all recorded by the Dutch police, as well as worrisome incidents that do not involve the juveniles themselves, but that are linked to the juvenile’s living address or to a co-occupant of the juvenile (in which the co-occupant can have any role as defined by the Dutch police). The recording of the latter group of worrisome incidents is important because it gives an indication of the extent to which a juvenile has to deal with risk factors in the home environment. Moreover, it makes the identification of problematic child-rearing situations possible at an early stage prior to situations of more serious concern (as in the case of supervision orders).

In sum, we defined the dependent variable (“care needs”) as the occurrence of a supervision order as imposed by a juvenile court judge and/or the occurrence of future worrisome incidents at the juvenile’s living address (involving child abuse, domestic violence, domestic strife, and/or sexual offensive behavior) within three years after the index incident occurred (i.e., from 2007 to 2010). The data about worrisome incidents were gathered from the Dutch police system, whereas data about supervision orders were requested at the Dutch Youth Care Agency. For juveniles who moved during the follow-up period, the incidents of the new living address were also retrieved. As for the independent variables, we searched in the police system for variables that were most consistent with predictors of child maltreatment mentioned in the literature. Police records of both the juvenile non-offenders as well as their co-occupants were retrieved from the Dutch police system. In addition, information on incidents at the juvenile’s living address was also requested. A complete list of all the independent variables that were retrieved from the police system can be found in Table 2.

**Table 2:** **Risk factors for future care needs (total sample;** ***N*** **= 3200)**

**Categorical independent variables**	**φ**
Born in The Netherlands (0 = No; 1 = Yes)	−0.081^***^
**Continuous independent variables**	**r**_**b**_
Current age	−0.060^*^
Age at first incident (involved in any role other than suspect)	−0.071^*^
Number of incidents (involved in any role other than suspect)	0.102^***^
Number of incidents (involved as victim)	−0.017
Number of incidents (involved as witness)	0.031
Number of incidents (involved as witness of violence)	−0.008
Number of incidents (involved as aggrieved person or reporter of an offense)	0.050
Number of incidents (involved and not having a specific role)	0.102^***^
Number of incidents (involved in any role not otherwise specified by the Dutch police)	0.088^**^
Number of incidents (involved in any role other than suspect), type of incident:	
Sex offenses without violence	0.097^***^
Sex offenses with violence	0.096^***^
Number of incidents in which another person than the juvenile was involved as a suspect and in which the juvenile was involved in any role other than suspect	0.107^***^
Number of incidents in which another person than the juvenile was most often involved as a suspect and in which the juvenile was involved in any role other than suspect	0.050
Number of incidents in which weapons were involved at the juvenile’s living address (the juvenile does not need to be involved in this incident)	0.157^***^
Number of incidents of sex offenses at the juvenile’s living address (the juvenile does not need to be involved in this incident)	**0.389** ^***^
Number of incidents of child abuse at the juvenile’s living address (the juvenile does not need to be involved in this incident)	**0.308** ^***^
Number of incidents of domestic violence at the juvenile’s living address (the juvenile does not need to be involved in this incident)	**0.534** ^***^
Number of incidents in which a co-occupant at the juvenile’s living address was a suspect	**0.470** ^***^
Number of incidents of child abuse in which a co-occupant at the juvenile’s living address was involved (in any role)	**0.490** ^***^
Number of incidents of domestic strife in which the juvenile and/or a co-occupant at the juvenile’s living address was a victim	**0.524** ^***^
Number of incidents of conflicts in which a co-occupant at the juvenile’s living address was a victim	**0.374** ^***^

A care need was defined as the occurrence of a supervision order as imposed by a juvenile court judge and/or the occurrence of future worrisome incidents at the juvenile’s living address (involving child abuse, domestic violence, domestic strife, and/or sexual offensive behavior) within a period of three years after the index incident.

Correlations ≥ .200 are in boldface to highlight the strongest associations.

*r*_*b*_ = biserial correlation; φ = phi-coefficient.

^*^ *p* < 0.05; ^**^ *p* < 0.01; ^***^ *p* < 0.001.

### Type of instrument

Within scientific literature, there has been a great deal of controversy regarding the most appropriate method for risk assessment. Meehl [19] was the first who introduced the issue of the clinical versus the actuarial approach to decision making and stated that the latter is superior to clinical judgment. Throughout the years, other researchers have shown that the actuarial approach to decision making equals or surpasses the clinical approach, and sometimes substantially [20-25]. It has even been suggested by Quinsey, Harris, Rice, and Cormier [26] that clinical judgment should be replaced completely by the use of actuarial instruments. At the same time, actuarial models have been criticized, for instance because purely actuarial predictions do not sufficiently take into account individual differences that can be crucial for an accurate prediction [27,28]. In addition, Scott and Resnick [29] mentioned that actuarial tools are rigid, lacking sensitivity to change, and cannot be generalized to populations other than the populations they were based on. More recently, the structured clinical judgment approach incorporating both empirically established risk factors and clinical judgment [30] has been proposed as the best approach to risk assessment [31,32]. However, several meta-analyses on the predictive validity of violence assessment instruments have found that structured clinical judgment and actuarial instruments perform at comparable levels of predictive accuracy [33,34]. Although actuarial instruments have been criticized, we believe that the actuarial approach to predicting future care needs of juvenile non-offenders is the most suitable for two reasons. First, police officers can assess several important risk factors for future care needs just by accessing information stored in the police registration system. Therefore, it can be assumed that an actuarial instrument solely based on this information may be promising in estimating the risk for future care needs in the initial stage of assessment. If this risk is considerable, police officers can refer the juvenile to the next link in the chain of youth care for a more thorough assessment that is conducted by specialized clinical professionals. Second, an actuarial instrument that is only based on information stored in a police system can be implemented in the same system, making it possible to automatically and rapidly screen large numbers of juveniles.

### Statistical analyses

First, we developed the Y-ACNAT-NO by conducting an Exhaustive Chi-squared Automatic Interaction Detector (Exhaustive CHAID) analysis. Tree classification methods like CHAID are very useful for gaining insight into profiles of youth with a high and low probability of care needs [35,36]. The purpose of CHAID is to create homogenous groups based on the value of a specific outcome variable (“care needs” in the current study), by splitting cases into two or more groups on the basis of several predictor variables [37,38]. This technique is particularly useful because the present study is exploratory rather than confirmatory, involves the association between several independent variables (which can interact with each other) and one dependent variable, and is not based on a strong theory concerning the relative importance of the independent variables in predicting the dependent variable [39]. We preferred CHAID analysis above logistic regression since the CHAID method is more sensitive in detecting interactions, and can be used to make decisions about how to combine categories within a variable to arrive at the simplest model [40,41]. Although CHAID and Exhaustive CHAID are very similar algorithms, the latter was preferred here, because Exhaustive CHAID performs a more thorough merging and testing of predictor variables than the regular CHAID algorithm [37]. Besides this, the results of a CHAID analysis are presented as a decision tree and easily interpretable, which may be an important aspect when police officers (or youth care workers) without substantial clinical expertise perform an initial assessment of care needs of juveniles.

The CHAID algorithm as applied in the current study involved repeatedly splitting (subsets of) the construction sample into two or more child nodes, beginning with the full construction sample. At each tree node, the best split was determined by merging any allowable pair of categories of the predictor variables until there was a statistically significant difference within the pair with respect to the target variable. The tree growing process stopped until no more statistically significant splits could be found or until the child nodes reached a minimum size (*n* = 20 in the present study). The result was a visual tree model in which the terminal nodes represent a number of “risk groups” with juveniles in each group having similar police records, and thus a similar risk for future care needs. When interpreting the tree model from top to bottom, it is possible to determine how the juveniles in the risk groups score on the variables that are part of the instrument. In the development of the current instrument, about 50% of the sample was used to build the model (construction sample) and about 50% of the sample was used to validate the instrument (validation sample).

Second, we assessed the discriminatory accuracy of the Y-ACNAT-NO by calculating AUC values in both the construction and validation sample, and by examining the sensitivity, specificity, and diagnostic odds ratio at various cutoff scores in the validation sample. An AUC value is a global and base rate resistant index of discrimination representing the probability that a randomly selected juvenile with care needs has a higher risk classification than a randomly selected juvenile without care needs. The sensitivity (or true positive rate) refers to the proportion of juveniles that is in need of care and actually tests positive on the Y-ACNAT-NO, whereas the specificity (or true negative rate) refers to the proportion of juveniles that is not in need of care and actually tests negative on the Y-ACNAT-NO. Ideally, a test should have high sensitivity and high specificity, but since sensitivity and specificity are inversely related, sensitivity is high when specificity is low and vice versa. To determine the cutoff score at which the sensitivity and specificity of the Y-ACNAT-NO are maximized we calculated the Youden index (*J*) which is defined as the maximum vertical distance between a ROC curve and the reference line (representing chance performance) and is calculated as *J* = maximum {sensitivity + specificity – 1} [42]. The cutoff point on the ROC curve that equals *J* (i.e., the point at which {sensitivity + specificity −1} is maximized) is regarded as the optimal cutoff point, but only under the assumption that sensitivity and specificity are of equal importance. The diagnostic odds-ratio is a base rate resistant measure for the discriminative power of the test and refers to the ratio of the odds of a true positive result relative to the odds of a false positive result. A higher diagnostic odds ratio indicates a better test performance and unlike an AUC value, it can take into account different cutoff scores. For a more thorough overview of these performance measures, see for instance the work of Singh [43].

Third, calibration of the Y-ACNAT-NO was assessed by first calculating the Brier score [44,45] and the Sanders-modified Brier score [46] in the construction and validation sample, which are single measures capturing both discrimination and calibration. The Brier score represents the difference between observed and predicted probabilities summed over all individual juveniles, whereas the Sanders-modified Brier score is summed over a discrete number of categories. The latter is of more interest in the present study, since the Y-ACNAT-NO classifies juveniles into different risk groups. Both scores range from 0 to 1 with 0 indicating perfect performance and .25 indicating that predicted outcomes agree with actual outcomes in 50 percent of the cases (i.e., chance). Spiegelhalter’s *Z* test [47] was performed to determine whether individual Brier scores were extreme, with significant results indicating that predicted and actual outcomes are not compatible. Next, we calculated a decomposition of the (Sanders-modified) Brier score designated as the *reliability-in-the-small* [45], which equals the squared error between the average predicted rate of care needs and the average observed rate of care needs in each risk category. A reliability-in-the-small of 0 indicates perfect calibration.

Finally, we calculated the positive predictive power, the negative predictive power, the number needed to detain, and the number safely discharged at various cut-off scores in the validation sample. The positive predictive power refers to the probability that a juvenile scoring above a particular cutoff score is correctly identified as someone with care needs, whereas the negative predictive power refers to the probability that a juvenile scoring below a particular cutoff score is correctly identified as someone without care needs. The number needed to detain is the number of juveniles judged to be at high risk for care needs who would need to be detained to prevent one actual case of care needs and the number safely discharged is the number of individuals judged to be at low risk for care needs who could be discharged before one case of care needs becomes actual. More information about these calibration measures can be found in the work of Singh [43].

Fourth, we examined whether the Y-ACNAT-NO outperforms the YO-CNAT [4] in the prediction of risk for future care needs of juvenile non-offenders. If so, maintaining a separate instrument for both juvenile offenders as well as juvenile non-offenders is justified. Otherwise, only the YO-CNAT should be retained and used for both groups of juveniles. For comparing the performance of both instruments, we first calculated the probability of future care needs for the juveniles in the current validation sample on the basis of the different risk groups of the YO-CNAT. Next, the ROC curve and AUC value of the Y-ACNAT-NO were compared to the ROC curve and AUC value of the YO-CNAT to assess differences in discriminatory accuracy between the two instruments. To assess differences in calibration, the (Sanders-modified) Brier score and the reliability-in-the-small of the Y-ACNAT-NO and the YO-CNAT were compared using the validation sample.

All analyses were performed using MedCalc for Windows, version 12.5 (MedCalc Software) or Stata/SE, version 12.0 (StataCorp, College Station, TX, USA) at a 95% significance level (two-sided). The method of DeLong et al. [48] was used for calculating the standard error of the area under the ROC curve, which was needed for constructing binomial exact confidence intervals of AUC values.

### Ethical approval

Formal Institutional Review Board approval for conducting the present study was not required, since this study involved secondary data analysis on de-identified data, which does not pose physical or psychological harm to participants. Accordingly, this study was ethically conducted based on the rules maintained by the Faculty Ethics Review Board (FMG-UvA) of the Faculty of Social and Behavioral Sciences of the University of Amsterdam in The Netherlands.

## Results

### Prevalences and risk factors

An overview of the prevalence of care needs within the three year period after the index incident is presented in Table 3 for both the construction and validation sample. In total, the percentage of juveniles that needed some form of care was 14.0% and 13.9%, respectively. No significant differences were found between the construction and validation sample in the prevalence of (specific types of) care needs. We conducted Z-tests for proportions to compare prevalences of care needs in the current sample to prevalences of care needs in the sample that was used to construct and validate the YO-CNAT. The results indicated that the prevalences of all types of care needs were significantly different between the two samples (*p* < .001, two-sided). Table 2 lists the association between all variables retrieved from the Dutch police system and whether or not juveniles were in need of care during the three year follow-up period.

**Table 3:** **Prevalence of care needs within three years after the index incident (** ***N*** **= 3200)**

	**Total sample**		**Construction sample**		**Validation sample**			
	**(** ***N*** **= 3200)**		**(** ***n*** **= 1549)**		**(** ***n*** **= 1651)**			
	***n*** ^**a**^	**%**	***n*** ^**a**^	**%**	***n*** ^**a**^	**%**	***χ*** ^**2**^ **(1)** ^**b**^	***Z*** ^**c**^
Number of juveniles with a supervision order	78	2.4	39	2.5	39	2.4	.029	7.982^***^
Number of juveniles associated with a worrisome incident^d^	405	12.7	198	12.8	207	12.5	.024	7.575^***^
of which: Incidents of domestic strife	120	3.8	67	4.3	53	3.2	2.453	6.021^***^
Incidents of domestic violence	295	9.2	146	9.4	149	9.0	.109	4.352^***^
Incidents of child abuse	88	2.8	50	3.2	38	2.3	2.229	3.503^***^
Incidents of sexual offensive behavior	103	3.2	60	3.9	43	2.6	3.734^+^	3.316^***^
Total number of juveniles with a care need	446	13.9	217	14.0	229	13.9	.004	16.169^***^

^a^Number of unique juveniles in the sample with a (specific) care need.

^b^Chi-square tests with Yates’ correction were conducted to determine differences between the construction and validation sample.

^c^ *Z*-tests for proportions were conducted to test for differences in the prevalences of care needs between the samples on which the Y-ACNAT-NO and the YC-NAT were based.

^d^Worrisome incidents are defined as incidents of domestic strife/violence, child abuse, and sexual offensive behavior that occur at the juvenile’s living address.

^+^ *p* < .10; ^***^ *p* < .001.

### Development of the Y-ACNAT-NO

**Figure 1:**
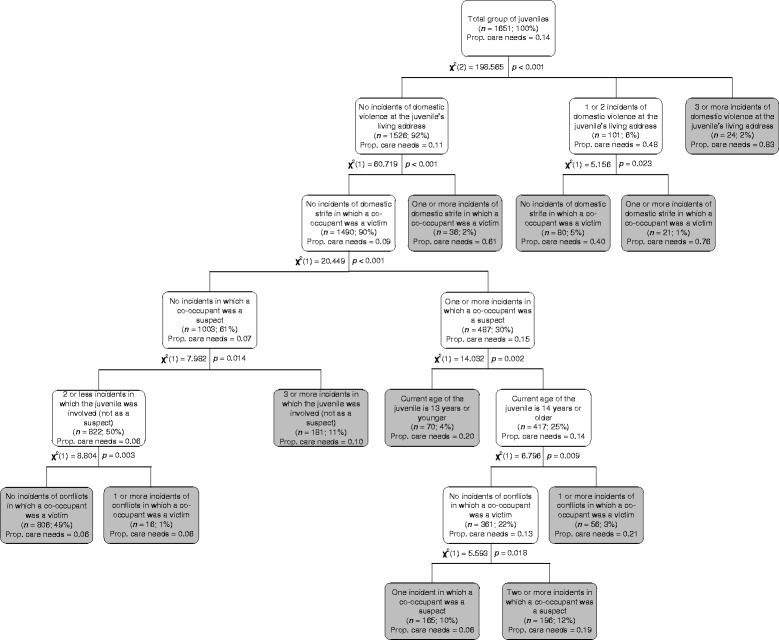
**Classification tree for the validation sample.** The grey shaded terminal nodes represent the eleven “risk groups” in which juveniles have similar scores on the variables that comprise the Y-ACNAT-NO and therefore the same risk for future care needs. *n* (%) = the number and percentage of juveniles that are classified in each tree node. Prop. care needs = proportion of juveniles with care needs.

Exhaustive CHAID analyses were conducted to develop the present actuarial screening tool (Y-ACNAT-NO). The variables mentioned in Table 2 were all included as independent variables and the dependent variable was whether or not juveniles had a care need (i.e., a juvenile placed under a supervision order and/or the occurrence of a worrisome incident at the juvenile’s living address) in the three year period after the index incident. The CHAID output for the validation sample is presented in Figure 1. To validate the CHAID model, the tree nodes that were derived in the construction sample were specified a priori in the validation sample rather than allowing the CHAID algorithm to build a new (and perhaps different) classification tree. The predictor variable for which the CHAID algorithm found the most significant split was the number of recorded incidents of domestic violence at the juvenile’s living address. Based on this variable, the total group of juveniles was divided into three different subgroups (see Figure 1). In the following step, these subgroups were further divided based on the next variable for which the most significant split could be found. This procedure of splitting subgroups was repeated until no further significant splits could be found or until the child nodes had reached a minimum size of *n* = 20. When the algorithm ended, the following six variables were part of the CHAID model: (1) the number of recorded incidents of domestic violence at the juvenile's living address; (2) the number of recorded incidents of domestic strife in which the juvenile and/or a co-occupant of the juvenile was a victim; (3) the number of recorded incidents in which a co-occupant of the juvenile was a suspect; (4) the number of recorded incidents in which the juvenile was involved (having any role other than suspect); (5) the current age of the juvenile; and (6) the number of recorded incidents in which a co-occupant of the juvenile was a victim of conflicts. As can be seen in Figure 1, eleven end nodes representing eleven different “risk groups” are part of the tree classification diagram. The risk for future care needs ranges from 0.06 in the lowest risk group to 0.83 in the highest risk group, meaning that approximately 6% of the juveniles in the lowest risk group will have care needs, whereas 83% of the juveniles in the highest risk group will have care needs. By interpreting the classification tree from top to bottom, it is possible to determine how the juveniles in the risk groups score on the six predictor variables that are part of the Y-ACNAT-NO.

### Predictive power of the Y-ACNAT-NO

The discrimination analyses produced an AUC of .804 (95% CI [.783, .823]) in the construction sample and an AUC of .764 (95% CI [.743, .784]) in the validation sample, which can be regarded as high according to Dolan and Doyle [49]. The sensitivity, specificity, and diagnostic odds ratio of the Y-ACNAT-NO are presented in Table 4 for various cutoff scores. Each cutoff score equals a risk for future care needs (i.e., the proportion of juveniles with care needs in a “risk group”), calculated by the CHAID algorithm in the construction sample.

**Table 4:** **Estimates of several performance measures for different cutoff scores of the Y-ACNAT-NO**

**Cutoff score (>)** ^**a**^	**Sensitivity (95% CI)**	**Specificity (95% CI)**	**Positive predictive power (95% CI)**	**Negative predictive power (95% CI)**	**Diagnostic odds ratio (95% CI)**	**NND (95% CI)**	**NSD (95% CI)**	**Youden index**
> .000	1.000 (.984 – 1.000)	.000 (.000 - .003)	.139 (.122 - .156)	-	-	7.194 (6.410 - 8.197)	-	.000
> .047	.799 (.741 - .849)	.535 (.508 - .561)	.217 (.189 - .246)	.943 (.925 - .958)	4.567 (3.252 – 6.414)	4.608 (4.065 - 5.291)	16.544 (12.333 – 22.810)	.334
> .053	.756 (.695 - .810)	.644 (.618 - .668)	.254 (.222 - .289)	.942 (.926 - .956)	5.575 (4.047 – 7.680)	3.937 (3.460 - 4.505)	16.242 (12.514 – 21.727)	.400
**> .117**	**.677 (.612 - .737)**	**.758 (.735 - .780)**	**.311 (.270 - .353)**	**.936 (.920 - .949)**	**6.564 (4.851 – 8.881)**	**3.215 (2.833 - 3.704)**	**14.625 (11.500 – 18.608)**	**.435**
> .128	.511 (.444 - .577)	.869 (.851 - .886)	.386 (.331 - .444)	.917 (.901 - .931)	6.942 (5.135 – 9.384)	2.591 (2.252 - 3.021)	11.048 (9.101 – 13.493)	.380
> .207	.507 (.440 - .573)	.880 (.862 - .896)	.404 (.347 - .464)	.917 (.901 - .931)	7.510 (5.539 – 10.182)	2.475 (2.155 - 2.882)	11.048 (9.101 – 13.493)	.387
> .257	.454 (.388 - .521)	.911 (.895 - .925)	.450 (.385 - .517)	.912 (.896 - .926)	8.484 (6.175 – 11.655)	2.222 (1.934 - 2.597)	10.364 (8.615 – 12.514)	.365
> .292	.393 (.329 - .460)	.950 (.937 - .961)	.559 (.479 - .637)	.907 (.891 - .921)	12.320 (8.624 – 17.602)	1.789 (1.570 - 2.088)	9.753 (8.174 – 11.658)	.343
> .439	.253 (.198 - .315)	.984 (.976 - .990)	.716 (.604 - .811)	.891 (.875 - .906)	20.631 (12.409 – 34.302)	1.397 (1.233 - 1.656)	8.174 (7.000 – 9.638)	.237
> .690	.087 (.054 - .132)	.997 (.993 - .999)	.833 (.626 - .953)	.872 (.854 - .887)	33.923 (11.482 – 100.223)	1.200 (1.049 - 1.597)	6.813 (5.849 – 7.850)	.084
>1.000	.000 (.000 - .016)	1.000 (.997 – 1.000)	-	.861 (.844 - .878)	-	-	6.194 (5.410 – 7.197)	.000

The presented measures pertain to the performance of the Y-ACNAT-NO in the validation sample.

^a^If a test score on the Y-ACNAT-NO (i.e., the probability of future care needs) is greater than the cutoff score, the test result is considered positive; otherwise, it is considered negative. The cutoff score with the highest Youden index [*J*] is indicated in bold.

CI = Confidence interval; NND = Number needed to detain; NSD = Number safely discharged.

The calibration analyses produced a Brier score of .0863 (Spiegelhalter’s *Z* = −.004, *p* = .502) and a Sanders-modified Brier score of .0881 in the construction sample (with the data grouped into eleven risk categories). The reliability-in-the-small was .0000 and the ratio of the *excess forecast variance* to the *minimum forecast variance* was 2.526, indicating that the observed variability of the predictions of the Y-ACNAT-NO was approximately 2.5 times the minimum variability that is necessary. According to Spiegelhalter [47], ratios exceeding 6.0 indicate substantial excess variation in risk predictions. In the validation sample, the calibration analyses produced a Brier score of .0964 (Spiegelhalter’s *Z* = 1.801, *p* = .072) and a Sanders-modified Brier score of .0976 (with the data grouped into eleven risk categories). The reliability-in-the-small was .0014, which corresponds to an average assessment error of 3.74% in risk estimates per risk category. The ratio of the excess forecast variance to the minimum forecast variance was 4.016. Overall, these results indicate an acceptable calibration of the Y-ACNAT-NO. The positive and negative predictive power, the number needed to detain, and the number safely discharged were examined for various cutoff scores and are presented in Table 4.

### Predictive value of the Y-ACNAT-NO for predicting specific types of care needs

We also examined whether the Y-ACNAT-NO is suitable for the prediction of specific types of care needs. Table 5 presents discrimination and calibration measures of the Y-ACNAT-NO for different dependent variables using the same CHAID output (as depicted in Figure 1). The discriminatory accuracy was high for the prediction of total worrisome incidents, child abuse, domestic strife, and sexual offensive behavior (with AUC values above .75 in the validation sample) and acceptable for the prediction of a supervision order and domestic violence (with AUC values between .70 and .75 in the validation sample). The calibration analyses produced acceptable (Sanders-modified) Brier scores for all dependent variables in the validation sample (with values < .100), but Spiegelhalter’s *Z* test indicated that predicted outcomes were only compatible with actual observations when either worrisome incidents or domestic violence was predicted. Furthermore, the reliability-in-the-small was only acceptable when the Y-ACNAT-NO was used for predicting worrisome incidents or domestic violence (with values < .0100).

**Table 5:** **Performance of the Y-ACNAT-NO when predicting specific types of care needs**

	**Construction sample**				**Validation sample**			
**Specific type of care needs**	**AUC (95% CI)**	**BS** ^**a**^	**SMBS**	**RIS**	**AUC (95% CI)**	**BS** ^**a**^	**SMBS**	**RIS**
Supervision order	.767^***^ (.745 - .788)	.0636^***^	.0628	.0389	.741^***^ (.719 - .762)	.0616^***^	.0604	.0378
Total worrisome incidents	.809^***^ (.788 - .828)	.0789	.0805	.0003	.758^***^ (.736 - .778)	.0899	.0904	.0023
Child abuse	.908^***^ (.892 - .921)	.0458^***^	.0482	.0230	.823^***^ (.804 - .841)	.0490^***^	.0497	.0299
Domestic violence	.790^***^ (.769 - .810)	.0721^**^	.0744	.0051	.740^***^ (.718 - .761)	.0797	.0821	.0093
Domestic strife	.937^***^ (.923 - .948)	.0416^***^	.0408	.0157	.870^***^ (.853 - .886)	.0458^***^	.0442	.0220
Sexual offensive behavior	.855^***^ (.837 - .872)	.0734^*^	.0715	.0452	.846^***^ (.828 - .863)	.0497^***^	.0500	.0277

^a^The statistical significance of individual Brier scores was determined by calculating the Spiegelhalter’s *Z* statistic, with significant values indicating extreme values. Non-significant results indicate better overall performance.

AUC = Area under the receiver operating characteristic curve.

CI = Binomial exact confidence interval.

BS = Brier score.

SMBS = Sanders-modified Brier score (with the data grouped into eleven risk categories).

RIS = Reliability-in-the-small.

^*^ *p* < .05; ^**^ *p* < .01; ^***^ *p* < 0.001.

### Comparing the predictive validity of the Y-ACNAT-NO and the YO-CNAT

**Figure 2:**
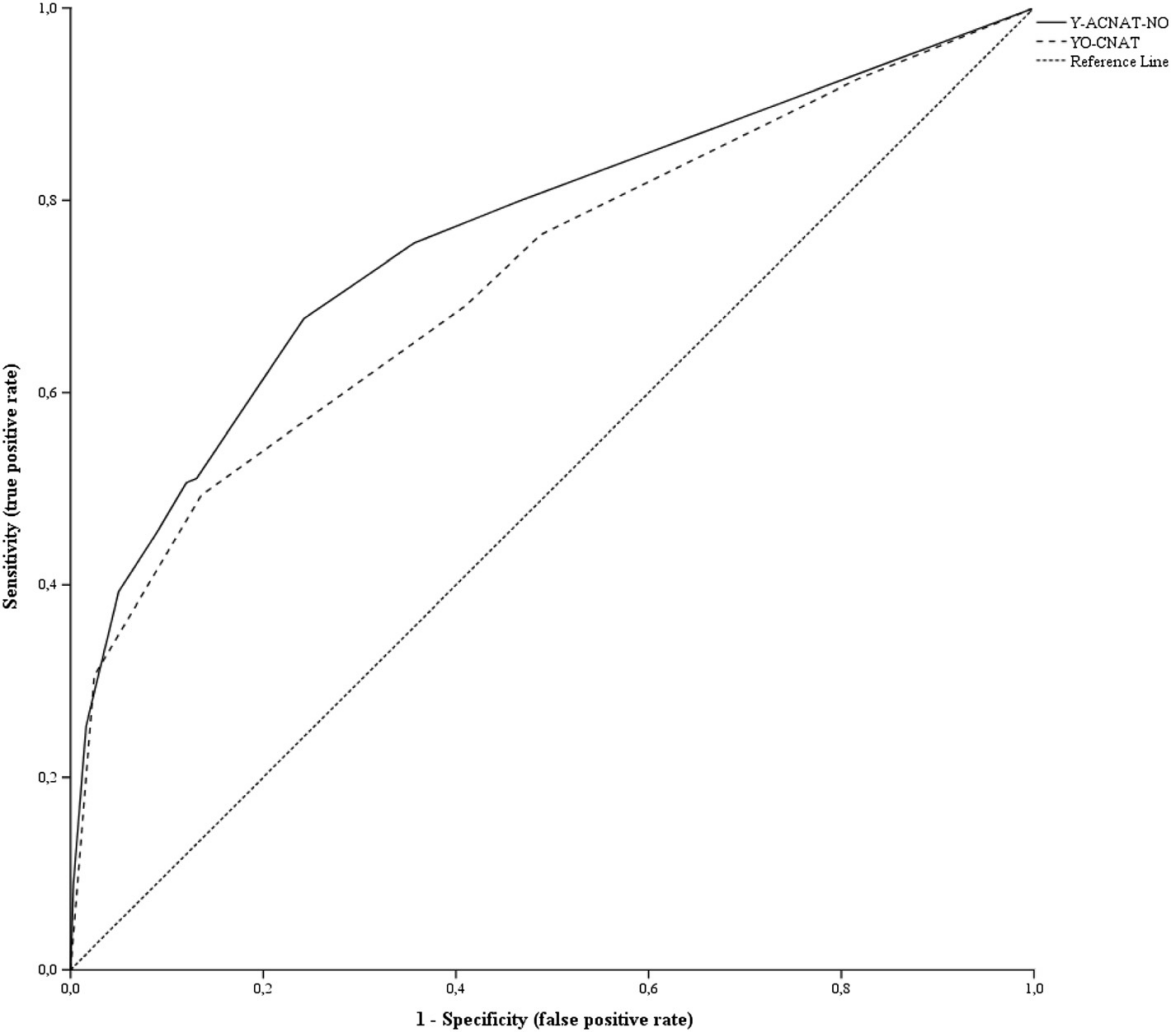
**Receiver operating characteristic curve analysis of the Y-ACNAT-NO and YO-CNAT.** Y-ACNAT-NO = Youth Actuarial Care Needs Assessment Tool for Non-Offenders; YO-CNAT = Youth Offender Care Needs Assessment Tool.

By comparing the predictive validity of the Y-ACNAT-NO to the predictive validity of the YO-CNAT we examined whether it is justified to maintain two different screening tools for the prediction of care needs, or that the YO-CNAT will suffice for predicting care needs of both juvenile offenders and juvenile non-offenders. As for the discriminatory accuracy, we first calculated the predicted probability of future care needs for each juvenile non-offender in the validation sample, by applying the YO-CNAT as described by Van der Put and Stams [4]. The predicted probabilities of future care needs based on the Y-ACNAT-NO that were also needed were already generated by the CHAID algorithm that was used for developing the Y-ACNAT-NO. Next, we created a ROC curve for both the Y-ACNAT-NO and the YO-CNAT and calculated the two AUC values. To verify if any difference between the AUC values of the two instruments were statistically significant, 95% confidence intervals were calculated using the method of DeLong et al. [48] and a significance test was performed. The results showed that the AUC value of the Y-ACNAT-NO (.764; 95% CI [.743 - .784]) was higher than the AUC value of the YO-CNAT (.722; 95% CI [.699 - .743]) and that the difference between these AUCs (.042; 95% CI [.011 - .073]) was statistically significant, *Z* = 2.662, *p* = .008. The ROC curves of both instruments are depicted in Figure 2.

To assess differences in calibration between the Y-ACNAT-NO and YO-CNAT, we first calculated several calibration measures for the YO-CNAT in the current validation sample. For the YO-CNAT, the Brier score was .1098 (Spiegelhalter’s *Z* = −7.064, *p* < .001), the Sanders-modified Brier score was .1175 (with the data grouped into five risk categories [4]), the reliability-in-the-small was .0105 (equal to an average assessment error of 10.25% in probabilistic estimates per risk category) and the ratio of the excess forecast variance to the minimum forecast variance was 5. A comparison of these calibration performance estimates to the calibration performance of the Y-ACNAT-NO (see above) indicates that the Y-ACNAT-NO outperforms the YO-CNAT.

## Discussion

Until recently, the development and validation of actuarial risk assessment instruments for predicting future care needs of juveniles did not receive much attention. Moreover, statistics on the predictive accuracy of risk assessment instruments for future child welfare involvement and/or child maltreatment are often not reported in published literature. Therefore, it is difficult to make a judgment about the predictive accuracy of the Y-ACNAT-NO relative to the predictive accuracy of other actuarial care needs assessment instruments that are specifically developed for juveniles. However, there are numerous actuarial instruments available that predict future delinquency or recidivism among juveniles and the discriminatory accuracy of the Y-ACNAT-NO (AUC = .764) compares favorably with the discriminatory accuracy of other instruments. For instance, Schwalbe [50] reported an average AUC value of .64 for assessment instruments predicting the risk for general recidivism and Fazel and colleagues [51] found a median AUC value of .66 for assessment instruments predicting the risk for general offending. In addition, the AUC value of the Y-ACNAT-NO meets the criterion of .75 mentioned by Dolan and Doyle [49] as a lower bound of high predictive validity. The calibration analyses indicated an average assessment error of 3.74% in risk estimates per risk category, which we regard as acceptable for an instrument that is to be used in the initial stage of assessment. For predicting specific forms of care needs, the Y-ACNAT-NO showed an acceptable discriminatory accuracy for all dependent variables (with AUC values of .740 or above), but calibration was only acceptable when worrisome incidents or domestic violence were predicted (with an average assessment error of less than 10% per risk category). Therefore, the Y-ACNAT-NO seems less suitable for the prediction of several specific care needs and future research is needed to improve the predictive validity by, for instance, examining additional predictor variables.

The choice of a suitable cutoff score depends largely on the consequences of false positive and false negative test results. For identifying the largest proportion of juveniles who are at risk for future care needs, the test should be highly sensitive at the expense of a less optimal specificity. If, however, further assessment is costly to society or may have unwanted (psychological) side effects, a high specificity is to be preferred. In deciding the optimal cutoff score, the result of a true high-risk juvenile (with actual care needs) not being referred for further assessment must be weighed against the result of a true low-risk juvenile (without actual care needs) who is being referred for further assessment. If the aim is to minimize the total number of inappropriate decisions (i.e., minimize the number of false positive and false negative test results), a cutoff score of .439 should be chosen. At this cutoff score, there are 24.8% false positives, 10.6% false negatives and 11.3% false decisions in total. However, if the consequences of false negatives are considered more serious than the consequences of false positives, a cutoff score of .128 can be chosen, at which the false negatives decrease (7.6%) at the cost of a considerable increase in false positives (57.9%). However, the percentage of total false decisions (16.8%) still seems acceptable at this cutoff score. Actuarial instruments have the advantage that numbers of false positives and false negatives can be calculated for all possible cutoff values so that an informed choice can be made by the police management about the cutoff score that best meets the requirements of the situation in which the instrument will be used.

The significant difference between AUC values of the YO-CNAT and Y-ACNAT-NO in combination with differences in the ROC curves of both instruments revealed that the Y-ACNAT-NO outperforms the YO-CNAT in situations where a high sensitivity (i.e., less false negatives) is to be preferred above a high specificity (i.e., less false positives). At most cutoff scores of the Y-ACNAT-NO the ratio between false negatives and false positives is optimal compared to the performance of the YO-CNAT at different cutoff scores, indicating the incremental discriminatory accuracy of the Y-ACNAT-NO. In addition, the results showed that the Y-ACNAT-NO is better calibrated than the YO-CNAT when the goal is to predict future care needs of juvenile non-offenders. We therefore believe that it is justified to maintain the Y-ACNAT-NO next to the YO-CNAT, which was developed for predicting future care needs of juvenile offenders.

The Y-ACNAT-NO can be used by the Dutch police in deciding whether juvenile non-offenders should be referred for further assessment. If a juvenile non-offender tests positive on the Y-ACNAT-NO (i.e., there is a considerable risk for future care needs), the police officer can decide to refer the juvenile to a youth care agency for further assessment. However, for an optimal decision it is important to note that the Y-ACNAT-NO should be used in addition to, and not instead of, the judgment of Dutch police officers. The test results of the Y-ACNAT-NO should not be regarded as perfect predictions of the risk for future care needs for at least two reasons. First, and in general, some degree of measurement error is inherent to risk assessment. Second, a number of important predictor variables for future care needs (see, for instance [17,18]) could not be part of the Y-ACNAT-NO, since information on these variables is not available in the Dutch police system. Consequently, false positive and false negative test results are inevitable at each possible cut-off score of the Y-ACNAT-NO. Therefore, the judgment of Dutch police officers remains important, even when an actuarial instrument is available that supports police officers in their decision about referring a juvenile for further assessment.

It should be stressed that the Y-ACNAT-NO cannot automatically be used in other countries than The Netherlands because of several reasons. The current screening instrument was constructed using only predictor variables that are stored in the Dutch police system, and it cannot be assumed that the same predictor variables are also stored in police systems in other countries. Moreover, the performance of the Y-ACNAT-NO at different cutoff scores are based on the prevalence of care needs in a Dutch sample. Because it is unlikely that exactly equal base rates of care needs are to be found in populations in different countries, the predictive validity (both discrimination and calibration) of the instrument needs to be examined in populations in which the instrument is to be applied. In addition, a cutoff score that is regarded as optimal in The Netherlands can be less optimal or even inappropriate for predicting the risk for future care needs of non-offending juveniles in other countries, making it necessary to determine an optimum cutoff score for each separate population. On the other hand, most predictor variables that constitute the Y-ACNAT-NO pertain to the number of previous episodes of maltreatment, which was found to be a key risk factor for the recurrence of child maltreatment in the systematic review of Hindley, Ramchandani and Jones [17]. We therefore think that results of CHAID analyses performed on samples from different countries than The Netherlands will not vary much, provided that data on the number of different types of worrisome incidents that occur in the direct living environment of the juvenile is available.

Several limitations of the current study should be noted. First, in developing the Y-ACNAT-NO, the aim was to construct a risk screening instrument that can be used by police officers without substantial clinical expertise, and that can easily be implemented in the police system. Therefore, we only used information stored in the Dutch police system for the construction of the instrument. There are, however, many other important predictors of child maltreatment and future care needs that are not recorded by the police. Future research on predicting care needs of juvenile non-offenders should therefore integrate both predictors derived from police records as well as other significant predictors to improve assessment or screening instruments. Second, a relatively small sample size was available to construct and validate the model. A more accurate model with better predictive accuracy may be obtained when samples of larger sizes were available. Third, the number of incidents as recorded by the police may be an underestimate of the actual number of incidents. It is likely that the actual number is greater, because not all incidents are reported or known to the police. Besides, it is often not an easy task to identify who was involved in an incident and in what specific role. These difficulties influence the validity of an actuarial instrument that is based on official police records.

## Conclusions

The present study extends risk screening research by showing that it was possible to develop an actuarial risk screening instrument with sufficient predictive value for the prediction of future care needs (i.e., problematic child-rearing situations) of juvenile non-offenders (i.e., juveniles who come into contact with the Dutch police but not in the role of a suspect). The Youth Actuarial Care Needs Assessment Tool for Non-Offenders can be administered by Dutch police officers and since it is solely based on police records and comprises only six items, it can easily be implemented in the Dutch police system, making the instrument efficient and cost-effective in the initial stage of risk assessment. By implementing the Y-ACNAT-NO, the Dutch police can play a more important role in timely identifying problematic child-rearing situations among juvenile non-offenders, which improves the chain of youth care organizations in The Netherlands significantly.
